# Use of dornase alfa in the paediatric intensive care unit: current literature and a national cross-sectional survey

**DOI:** 10.1136/ejhpharm-2020-002507

**Published:** 2020-10-29

**Authors:** Bibiche den Hollander, Rosalie S N Linssen, Bart Cortjens, Fardi S van Etten-Jamaludin, Job B M van Woensel, Reinout A Bem, the Dutch Collaborative PICU Research Network

**Affiliations:** 1 Pediatric Intensive Care Unit, Emma Children's Hospital, Amsterdam UMC, location AMC, Amsterdam, The Netherlands; 2 Research Support, Medical Library, Amsterdam UMC Location AMC, Amsterdam, North Holland, The Netherlands

**Keywords:** critical care, evidence-based medicine, pediatrics, pulmonary medicine, quality of health care

## Abstract

**Objectives:**

Airway mucus obstruction is a major challenge in children admitted to the paediatric intensive care unit (PICU). We aimed to evaluate the evidence and contemporary use of the mucolytic medication dornase alfa for non-cystic fibrosis conditions in the PICU.

**Methods:**

(1) We performed a systematic review with searches in PubMed, EMBASE, and the Cochrane Library. *Study selection:* for quality assessment and data synthesis, we included only randomised controlled trials (RCTs) that compared dornase alfa to standard care or placebo in critically-ill paediatric patients (<18 years of age) in the PICU. However, non-randomised controlled studies and case series are also discussed. *Data extraction:* data were extracted independently by multiple reviewers using data extraction forms. The primary outcome was duration of mechanical ventilation. *Data synthesis:* The GRADE approach was used for quality assessment. No meta-analysis could be performed. (2) A national cross-sectional survey among all seven PICUs in the Netherlands was also performed.

**Results:**

The systematic review yielded only one RCT, comparing dornase alfa with normal saline in children after cardiac surgery. In this study, dornase alfa led to a reduction in duration of mechanical ventilation by approximately 1 day (36% reduction). In addition, we found nine retrospective observational and case studies. The survey revealed high current use of dornase alfa in Dutch PICUs: 42% of the respondents reported prescribing dornase alfa at least once every week. Only 4% of the respondents reported having access to a local PICU dornase alfa protocol.

**Conclusions:**

The off-label use of dornase alfa in the PICU is frequent without strong evidence or local protocols, highlighting the need for further research on the effectiveness of this mucolytic agent.

## Introduction

Airway obstruction resulting from a combination of mucus hypersecretion, increased mucus viscosity and decreased mucociliary clearance poses a major challenge in the care for critically-ill children with respiratory disease in the paediatric intensive care unit (PICU).^1^ The presence of mucus plugs carries the risk of atelectasis and nosocomial (eg, ventilator-associated) pneumonia, as well as prolonged need for respiratory support.^2 3^ This is particularly true in young children due to small sized airways in combination with limited respiratory muscle strength and possibilities for collateral ventilation.^4^ It is likely that the effectiveness of mucoactive medications aimed at preventing and diminishing airway mucus obstruction is highly affected by factors that impair normal mucociliary function and cough clearance such as endotracheal intubation, application of positive airway pressure ventilation and sedatives with or without neuromuscular blocking agents.

Mucus viscosity is substantially increased by the presence of extracellular DNA,^5^ which is released in the airways from dead airway epithelial cells and recruited leucocytes. The peptide mucolytic agent dornase alfa (Pulmozyme) depolymerises the mucus gel network by cleaving DNA and hence reduces mucus viscosity. In children, dornase alfa is currently only registered as a treatment for cystic fibrosis (CF).^6^ However, despite its relatively high costs, off-label use has been widely reported in the management of a variety of paediatric respiratory disorders, although with limited success.^7 8^ Interestingly, in recent years there has been renewed attention paid to the potential beneficial effects of dornase alfa, in particular in critically-ill patients, related to the discovery of neutrophil extracellular traps (NETs), which form an important source of extracellular DNA with potent cytotoxic and oxidative properties in the airways and lungs.^9–13^

The extent of off-label use of dornase alfa in non-CF patients in the PICU is largely unknown. To provide further insight, we performed a systematic review of the literature to establish the current evidence and carried out a national survey to determine the contemporary use of dornase alfa in PICUs in the Netherlands.

## Methods

### Systematic review

We first performed a systematic review of the literature following the Cochrane collaboration principles and quality assessment, in accordance with the Preferred Reporting Items for Systematic Reviews and Meta‐Analyses (PRISMA) statement (see checklist online supplemental material).^14^ Of note, this systematic review is connected to a previous independent, broader systematic review protocol as submitted to PROSPERO (ID: CRD42019132634).

10.1136/ejhpharm-2020-002507.supp1 Supplementary data

#### Primary and secondary outcome indicators

Our primary objective was to evaluate the effectiveness of dornase alfa in critically-ill paediatric patients compared with standard care or placebo in reducing the duration of invasive mechanical ventilation. Secondary outcomes included: duration of PICU stay (days), atelectasis resolution or improvement on chest X-ray, re-intubation rate, mortality, respiratory system mechanics measurements (airway pressures and resistance, lung compliance, respiratory system compliance), oxygenation/ventilation indices, and adverse events.

#### Selection criteria

For data synthesis and quality assessment, we only included randomised controlled trials (RCTs) that compared dornase alfa to standard care or placebo in critically-ill paediatric patients (<18 years of age) admitted to the PICU. However, observational studies and case studies are also reported unless only available in abstract form. Studies with CF patients, laboratory or animal studies, studies that were not available in English and studies that were not carried out in a PICU were excluded from this review.

#### Data collection and analysis

We performed a final search on 21 July 2020 for relevant studies in the electronic databases Pubmed, EMBASE, and Cochrane Library. Databases were searched from inception to the dates as stated using search terms as provided in the online supplemental material.

#### Selection of studies

**Figure 1:**
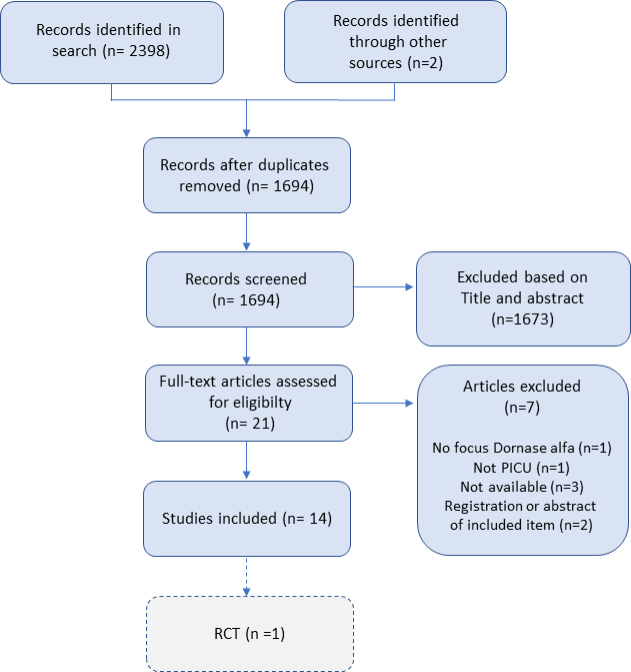
Flow diagram of included studies from the systematic search on dornase alfa treatment for non-CF conditions in critically-ill children in the PICU. CF, cystic fibrosis; PICU, paediatric intensive care unit; RCT, randomised controlled trial.

Two review authors (BH and RL) removed duplicate reports and screened titles and abstracts identified by the search for eligibility and coded them as “retrieve” (eligible or potentially eligible/unclear) or “do not retrieve”. Review authors BH and RL independently checked those marked as eligible, potentially eligible or ineligible against the inclusion criteria. We retrieved the full-text of eligible trial reports or publications and four review authors (BH, RL, BC and RB) independently screened the full-text articles and identified studies for inclusion, recording reasons for exclusion of ineligible studies. The references of relevant articles were checked for potentially eligible studies.^15^ The results from the screening and eligibility process are shown in a flow diagram (figure 1) in accordance with PRISMA guidelines.^14^

#### Data extraction and management

Data were captured using a standardised data extraction form. Study information was extracted on the design and setting of the study, inclusion and exclusion criteria, participant characteristics (including sex, age, underlying conditions), nature of the intervention(s) in each group (eg, dose, route of administration, concomitant therapies), time-points of measurements, and outcomes as described above. Any disagreements were resolved through discussion or, if required, with consultation of the other review authors.

#### Assessment of risk of bias in included studies

For eligible RCTs, four review authors (BH, RL, BC and RB) independently used the Cochrane risk of bias tool for randomised trials.^16^ Each potential source of bias was marked as high, low or unclear risk of bias with justification.

#### Data synthesis

Data are presented in a summary of findings table. We determined an overall grading of the evidence for the outcomes using the principles of the GRADE system to determine the strength of the evidence. The GRADE approach assesses the quality of evidence into one of four grades: high, moderate, low or very low.^17^

For binary variables, data were calculated as relative risk (RR) with 95% confidence intervals (95% CI). Data for continuous variables were calculated as mean differences (MD with 95% CI) when applicable. Medians of continuous variable were presented for each group, as no median difference could be calculated.

### Cross-sectional survey

In July 2019 an online, anonymous survey containing questions pertaining to the current use (indications and aetiologies, frequency of prescription, dosage, route of administration, safety, need for evidence) of dornase alfa was distributed via Limesurvey to the full medical staff and fellows (n=99) of all seven PICUs in the Netherlands (~4000 admissions per year). Email addresses were retrieved via the Dutch Paediatrics Association (NVK), section Paediatric Critical Care. The survey was created and distributed following current available recommendations where possible and appropriate.^18^ Survey questions (see online supplemental material) were created based on current available literature and the aims of this study. No pre-existing surveys or questionnaires were used. The survey was accompanied by a cover letter explaining the rationale of the survey. A waiver from the local ethical committee for the distribution of the survey was obtained (W20_404). The online survey was tested for correct functioning by the medical research support institute of the Amsterdam UMC and the authors. A reminder was sent to non-responders after 7 weeks, but reasons for non-responding were not recorded. Data were analysed with descriptive measures.

10.1136/ejhpharm-2020-002507.supp2 Supplementary data

## Results

### Literature review

The search on 21 July 2020 yielded a total of 2398 results of which only one study was an RCT fulfilling inclusion criteria, while 11 were observational or case studies (figure 1).^19–30^ Four of these studies were available in (conference) abstract form only and are not discussed further due to lack of essential details. One additional retrospective study and one additional case study was identified through checking the references of relevant studies.^31 32^ An overview of the 10 studies on dornase alfa use in the PICU can be found in table 1.

**Table 1:** All studies reporting on paediatric and non-CF patients in the PICU

First author, year of publication	Study design	Geography	Patient population	Intervention (I) route and control (C) treatment	Main findings according to primary and secondary systematic review outcomes
Riethmueller 2006*^21^	RCT	Germany, PICU	Invasive MV after cardiac surgery (n=88) Age: 0–2 years	I=dornase alfa by tracheal instillation C=0.9% saline	Shorter duration of MV (2.2 vs 3.4 days, p=0.043), length of stay and incidence of atelectasis. Similar re-intubation rates
Hendriks 2005^31^	Retrospective observational study, before-after analysis	The Netherlands, PICU and medium care	Patients with atelectasis (n=30, but n=25 on PICU with n=16 on MV) Age: 1.6 years median	I=dornase alfa by nebulisation or tracheal instillation	Duration of MV <6 days in 12/16 patients. Improvement in chest X-ray scores and oxygenation/ventilation indices (eg, pCO_2_, FiO_2_) before-after (p<0.01). Transient desaturation (n=3)
Riethmueller 2009^22^	Retrospective observational study with historical control group, before-after analysis	Germany, PICU	Invasive MV with atelectasis (intervention group, n=46) or after cardiac surgery (historical control group from ref 21, n=17) Age: 0.48 years median	I=dornase alfa by tracheal instillation C=NaCl 0.9%	Improvement of chest X-ray atelectasis in 67% of patients receiving dornase alfa, vs 6% in controls. Improvement of respiratory mechanics and oxygenation (airway pressures, FiO_2_)
Prodhan 2009^20^	Retrospective observational study, before-after analysis	USA, PICU	Invasive MV with atelectasis and congenital cardiac disease (n=38) Age: 3.5 months median	I=dornase alfa for <14 days by nebulisation	Improvement of chest X-ray atelectasis (p<0.05). No change in respiratory mechanics/ventilation indices, small decrease in FiO_2_ (p<0.001). No adverse events
Ozturk 2014^19^	Retrospective observational study, case-control and before-after analysis	Turkey, PICU	Postoperative atelectasis after congenital heart surgery (n=41) Age: 25.5 days median (dornase group) and 50.0 days median (control group)	I=dornase alfa by tracheal instillation or nebulisation C=conventional medication (eg, albuterol)+chest physiotherapy	Improvement chest X-ray atelectasis (p<0.01) and oxygenation indices (pO_2_, p=0.04) in dornase alfa group vs non-significant in control group
Greally 1995^32^	Case report	Ireland, PICU	Invasive MV for status asthmaticus with complete lung atelectasis in 8-year-old female	I=dornase alfa by tracheal instillation with bronchoscopy	Complete resolution atelectasis with fast clinical improvement (not specified). No adverse effects
Durward 2000^25^	Case report	Canada, PICU	Invasive MV for status asthmaticus with atelectasis in 7-year-old male	I=dornase alfa by tracheal instillation with bronchoscopy	Resolution atelectasis and fast improvement in respiratory mechanics and ventilation/oxygenation indices. No adverse effects
Patel 2000^26^	Case report	UK, PICU	Invasive MV for refractory status asthmaticus in 3-year-old male	I=dornase alfa by tracheal instillation with bronchoscopy	Fast improvement in respiratory mechanics and ventilation indices (pCO_2_). No adverse effects
Merkus 2001^24^	Case report	The Netherlands, PICU	Infants with severe RSV bronchiolitis (n=5, with n=3 with invasive MV) 2–29 weeks old	I=dornase alfa by nebulisation	Differential improvement in chest X-ray/atelectasis, improvement in oxygenation/ventilation indices. No adverse effects
Manna 2003^23^	Case report	UK, PICU	Invasive MV with plastic bronchitis and acute chest syndrome (sickle cell disease) in 7-year-old male	I=dornase alfa by tracheal instillation with bronchoscopy/chest physiotherapy	Improvement in chest X-ray, removal of bronchial casts, improvement in ventilation indices

*More detailed information on the RCT of Riethmueller 2006 is reported in the main results and online supplemental tables 1 and 2.

CF, cystic fibrosis; FiO_2_, fraction of inspired oxygen; MV, mechanical ventilation; pCO_2_, partial pressure of carbon dioxide; PICU, paediatric intensive care unit; pO_2_, partial pressure of oxygen; RCT, randomised controlled trial; RSV, respiratory syncytial virus.

10.1136/ejhpharm-2020-002507.supp3 Supplementary data

10.1136/ejhpharm-2020-002507.supp4 Supplementary data

The use of dornase alfa in the PICU was studied for the treatment of atelectasis in critically-ill children receiving invasive mechanical ventilation,^19–22 31^ and more specifically in patients with bronchiolitis,^24^ asthma^25 26^ and plastic bronchitis.^23^ In the one RCT that was found,^21^ intratracheal dornase alfa was compared with normal saline twice daily in children after cardiac surgery (n=88). This study found no difference in re-intubation rates (primary outcome of that study), but observed a reduction in duration of mechanical ventilation by approximately 1 day (52 vs 82 hours, 36% reduction, p<0.05) in favour of dornase alfa (for a summary of findings see online supplemental table 1). Similar trends were found for length of stay in the PICU (dornase alfa shortened PICU stay by 25%, 95% CI 6% to 42%, corrected for age), while the odds ratio of atelectasis (by blinded assessment of chest X-rays) during dornase alfa treatment compared with saline was 0.27 (95% CI 0.08 to 0.84). While the overall quality of evidence of this study was good, there was a potential for publication bias or threat to the study validity based on financial support from the manufacturer of dornase alfa without prior study protocol registration (risk of bias assessment, online supplemental table 2).

In addition, four retrospective studies with small sample sizes (n<50) in mechanically ventilated patients described improvement of atelectasis, chest X-ray scores, respiratory and heart rate, and gas exchange/ventilator parameters, but all with different end points between studies.^19 20 22 31^ We found five case series/reports describing anecdotal improvements of (refractory) atelectasis and gas exchange/ventilator parameters with the use of dornase alfa in children.^23–26 32^ None of the above studies reported serious adverse effects associated with the use of dornase alfa.

### Survey

**Figure 2:**
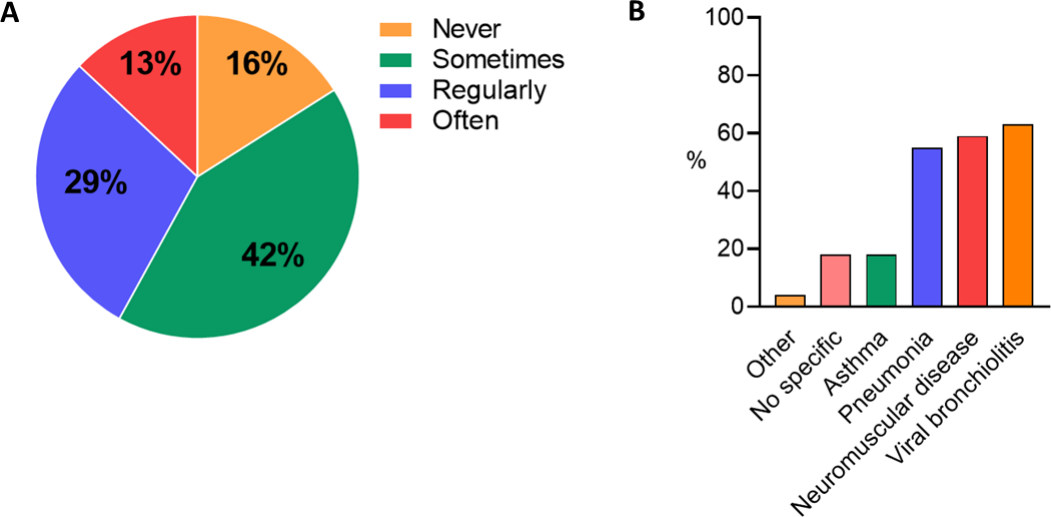
(A) Percentage of respondents that reported never, sometimes (<1 x per week), regularly (1 x per week) or often (>1 x per week) prescription of dornase alfa in patients admitted to the PICU. (B). Percentage of respondents reporting use of dornase alfa in non-CF aetiologies in the PICU. CF, cystic fibrosis; PICU, paediatric intensive care unit.

The survey response rate was 46% (42 intensivists, three fellows). This included responses from all seven PICUs, with a mean±SD of 14.3±5.5% of the respondents per centre. Respondents from all but one PICU reported prescribing dornase alfa for non-CF conditions, and 42% reported frequent use (≥1 times per week) (figure 2). This was despite most respondents (96%) reporting the lack of, or were unaware of, a local hospital or PICU protocol/guideline for the use of dornase alfa in non-CF patients. Primary reasons for the use of dornase alfa in non-CF patients were X-ray confirmed atelectasis (83%), or nurse-reported increased mucus viscosity or mucus evacuation problems (80%). The most common non-CF diagnosis in which dornase alfa was prescribed was viral bronchiolitis (63%), followed by neuromuscular disease and pneumonia (figure 2). Reported use of dornase alfa was higher for children receiving invasive as compared with non-invasive mechanical ventilation (96% vs 61%). By far the most common route of administration was via nebulisation, but occasional direct intratracheal instillation through the endotracheal tube was also reported by 40% of the respondents. Mild and transient side effects were reported to occur in up to 25% of the patients, most commonly (13%) being immediate respiratory symptoms (eg, transient desaturation) following mucus plug mobilisation. In total, 84% of the respondents stated that further research on the effectiveness of dornase alfa in the PICU, specifically related to duration of respiratory support as a primary outcome, and in particular patient groups including severe viral bronchiolitis, neuromuscular disease and confirmed atelectasis, is necessary.

## Discussion

The main finding from our systematic review of the literature is that the evidence for effectiveness of dornase alfa treatment for airway mucus obstruction in critically-ill children with non-CF diseases in the PICU is scarce, comprising only one RCT in addition to several observational/case studies. This importantly contrasts with the main finding of our national, multicentre survey reporting a remarkable frequent off-label use of dornase alfa in non-CF conditions in the PICU in the Netherlands, despite a lack of local protocols.

To our knowledge, this systematic review is the first to study the current evidence for dornase alfa specifically in the PICU. While several retrospective and case studies suggest beneficial effects of dornase alfa on resolution of atelectasis and/or clinical indices were identified, there is only one RCT of good quality which showed shortening of duration of mechanical ventilation (primary outcome in this systematic review), but only in a specific age and disease population. This paucity of data in critically-ill children, who are in general highly prone to develop respiratory failure following airway mucus obstruction related to their small airway diameter,^1–3^ underscores the need for further randomised controlled testing in superiority or non-inferiority trials. Interestingly, while airway mucus obstruction has been identified as one of the major challenges in the management of critically-ill patients,^33^ the adult ICU literature shows a similar lack of evidence-based recommendations on mucoactive agents, including dornase alfa.^34^ Possibly, the recent renewed increase in interest regarding dornase alfa in critically-ill patients, following the implication of NETs as a major source of extracellular DNA networks in the airways and lungs during various diseases,^9–13^ will facilitate the planning and design of future RCTs.

Despite the limited evidence for effectiveness as well as reported lack of local protocols for dornase alfa, current reported use of this mucolytic agent in PICUs in the Netherlands was high. These findings are in line with a recent single centre PICU study from the US reporting major usage of dornase alfa, which was associated with high costs of up to US$150 000 per year.^35^ In an era with much need for applying meaningful critical care, randomised controlled testing in superiority or non-inferiority trials comparing dornase alfa with normal saline administration in the treatment of airway mucus obstruction is warranted. Interestingly, pharmacist-guided implementation of dornase alfa protocols in the PICU has been shown to reduce the use and costs of dornase alfa by approximately 75%, without increasing PICU length of stay.^35^ This further underscores the potential benefit of close involvement of pharmacists in the multidisciplinary PICU environment to guide protocols and ensure appropriate prescribing.^36^

Following the frequent use of dornase alfa despite limited scientific evidence, potential safety issues, in addition to the associated costs as discussed above, should also be addressed. Luckily, dornase alfa is in general well tolerated, with mild adverse effects such as voice alteration or rash reported in large meta-analyses in patients with CF.^6^ Similar findings are found in young children.^37^ Likewise, all studies from our systematic review report no serious adverse events related to dornase alfa treatment in PICU patients. However, transient desaturation in mechanically ventilated children is reported in several studies, as well as in our survey, by PICU clinicians. Such an adverse effect likely results from breakdown of large mucus plugs, leading to scattering of mucus into more distal airways and the alveolar compartment, and should certainly be taken into account when using dornase alfa in the PICU. Furthermore, lysis of NETs in the lungs by dornase alfa may contribute to the release and spreading of captured pathogens, although so far this remains a largely theoretical concern.^11^

Based on our combined systematic review and survey results, we have identified the need for future randomised controlled testing of (cost-)effectiveness of dornase alfa in the PICU. Given the notorious heterogeneous patient population coupled with a challenge to obtain large sample sizes in PICU research, RCTs may be focused on largely homogenous diseases such as severe viral bronchiolitis, as was also identified as a priority in our survey. Furthermore, based on our survey, we propose the duration of invasive mechanical ventilation as the primary clinical outcome in future RCTs (eg, within a core outcome set), instead of focusing on (chest X-ray confirmed) atelectasis or oxygenation/ventilation indices. Duration of mechanical ventilation needs standardising of weaning/extubation protocols, but given the general low PICU mortality which is often unrelated to severity of respiratory disease,^38^ this clinical outcome may be more appropriate than mortality or related composite outcomes such as ventilator-free days. Additionally, specific future areas of interest are the route of administration (nebulisation vs direct endotracheal) and, importantly, direct assessment of mucus rheological changes following mucolytic treatment with dornase alfa, as this should provide better insight into the biophysical effects of dornase alfa on mucus.

A limitation of our systematic review was that only one (industry sponsored) RCT was found, focusing on one specific patient PICU population, which hampered our conclusions. In addition, a limitation of our survey study was the moderate response rate, although this was certainly reasonable when considering nationwide surveys in general. This may have caused bias by reporting higher use of dornase alfa. However, responses from all PICUs were documented, which strengthens our findings.

In conclusion, based on a systematic review of the literature, there is insufficient evidence to recommend dornase alfa as a routine treatment for airway mucus obstruction or atelectasis in critically-ill children with non-CF diseases in the PICU. Despite this paucity of data and lack of local protocols, the current use of dornase alfa in PICUs in the Netherlands is high. Given the clinically relevant and challenging treatment of airway mucus obstruction in critically-ill children, but also the potentially high costs associated with dornase alfa use, further research on the effectiveness of dornase alfa in the PICU is needed.

## Data Availability

Data are available upon reasonable request. All data relevant to the study are included in the article or uploaded as supplementary information.
