# Supplementary material for: Use of dornase alfa in the paediatric intensive care unit: current literature and a national cross-sectional survey
Source: Eur J Hosp Pharm. 2020 Oct 29;29(3):123–8. doi: 10.1136/ejhpharm-2020-002507 (PMC9047925; doi:10.1136/ejhpharm-2020-002507)
Supplement: Supplementary data [file ejhpharm-2020-002507supp001.pdf]

# Supplemental Material

**Search strategies**

**PubMed:**

("dornase alfa" [Supplementary Concept] OR "Deoxyribonuclease I"[Mesh] OR pulmozyme[tiab] OR dornase[tiab] OR DNase[tiab] OR rhDNase[tiab] OR deoxyribonuclease[tiab]) AND ("Intensive Care Units, Pediatric"[Mesh] OR "Child"[Mesh] OR "Adolescent"[Mesh] OR "Pediatrics"[Mesh] OR "Infant"[Mesh] OR PICU[tiab] OR PICUs[tiab] OR child*[tiab] OR pediatric*[tiab] OR paediatric*[tiab] OR adolescent*[tiab] OR teen*[tiab] OR puber*[tiab] OR pubescen*[tiab] OR infant*[tiab] OR youth[tiab] OR schoolchild*[tiab] OR juvenile*[tiab] OR minors[tiab] OR kid[tiab] OR kids[tiab]) NOT ("Letter"[Publication Type] OR "Editorial"[Publication Type] OR "Comment"[Publication Type] OR letter[ti] OR editorial[ti]) NOT (("Animals"[Mesh] OR "Animal Experimentation"[Mesh] OR "Models, Animal"[Mesh] OR rat[tiab] OR rats[tiab] OR mice[tiab] OR mouse[tiab] OR dog[tiab] OR dogs[tiab] OR pig[tiab] OR pigs[tiab] OR cow[tiab] OR cows[tiab] OR monkey[tiab] OR monkeys[tiab] OR horse[tiab] OR horses[tiab]) NOT ("Humans"[Mesh] OR human*[tiab]))

**EMBASE (Ovid):**

Embase Classic and Embase 1947 to 2020 July 17

| # | Searches |
|---|---|
| 1 | dornase alfa/ or deoxyribonuclease I/ |
| 2 | (pulmozyme or dornase or DNase or rhDNase or deoxyribonuclease).ti,ab,kw. |
| 3 | 1 or 2 |
| 4 | pediatric intensive care unit/ or exp child/ or pediatrics/ or juvenile/ or adolescent/ or exp infant/ |
| 5 | (PICU or PICUs or child* or pediatric* or paediatric* or adolescent* or teen* or puber* or pubescen* or infant* or youth or schoolchild* or juvenile* or minors or kid or kids).ti,ab,kw. |
| 6 | 4 or 5 |
| 7 | 3 and 6 |
| 8 | letter/ or editorial/ or note/ or (letter or comment or editorial).ti. |
| 9 | 7 not 8 |
| 10 | (exp animal/ or exp animal experiment/ or exp animal model/ or (rat or rats or mice or mouse or dog or dogs or pig or pigs or cow or cows or monkey or monkeys or goat or goats or horse or horses).ti,ab,kw.) not (human/ or human*.ti,ab,kw.) |
| 11 | 9 not 10 |

**Cochrane Library (Issue 7 of 12, July 2020)**

| ID | Search Hits |
|---|---|
| #1 | (pulmozyme or dornase or DNase or rhDNase or deoxyribonuclease):ti,ab,kw |
| #2 | MeSH descriptor: [Deoxyribonuclease I] explode all trees |
| #3 | #1 or #2 |
| #4 | (PICU or PICUs or child* or pediatric* or paediatric* or adolescent* or teen* or puber* or pubescen* or infant* or youth or schoolchild* or juvenile* or minors or kid or kids):ti,ab,kw |
| #5 | MeSH descriptor: [Intensive Care Units, Pediatric] explode all trees |
| #6 | MeSH descriptor: [Child] explode all trees |
| #7 | MeSH descriptor: [Adolescent] explode all trees |
| #8 | MeSH descriptor: [Infant] explode all trees |
| #9 | #4 or #5 or #6 or #7 or #8 |
| #10 | #3 and #9 |
