# Supplementary material for: Use of dornase alfa in the paediatric intensive care unit: current literature and a national cross-sectional survey
Source: Eur J Hosp Pharm. 2020 Oct 29;29(3):123–8. doi: 10.1136/ejhpharm-2020-002507 (PMC9047925; doi:10.1136/ejhpharm-2020-002507)
Supplement: Supplementary data [file ejhpharm-2020-002507supp002.pdf]

Supplemental Material: Survey Questions
Current use

1. **Are the patients on your PICU treated with dornase alfa (Pulmozyme)?**
   Choose one of the following answers
   ☐ No
   ☐ Yes, sometimes (<1/week)
   ☐ Yes, regularly (1/week)
   ☐ Yes, often

2. **Please provide an estimation of the number of times you prescribed dornase alfa (Pulmozyme) in the last 6 months.**
   Only numbers may be entered in this field
   ☐

3. **Are patients <u>without</u> cystic fibrosis also treated with dornase alfa (Pulmozyme)?**
   ☐ Yes ☐ No

4. **If yes, for which indication do you (sometimes) prescribe dornase alfa (Pulmozyme) in patients <u>without</u> cystic fibrosis?**
   Check all that apply
   ☐ Clinically supsected atelectasis
   ☐ X-ray confirmed atelectasis
   ☐ Nurse-reported high mucus viscosity or mucus evacuation problems
   ☐ Other: ______

5. **If yes, for which patient groups do you (sometimes) prescribe dornase alfa (Pulmozyme) in patients <u>without</u> cystic fibrosis**
   Check all that apply
   ☐ No specific patient group
   ☐ Viral bronchiolitis
   ☐ Pneumonia
   ☐ Asthma
   ☐ Neuromuscular patients
   ☐ Other: ______

6. **If yes, what dose regimen do you prescribe to patients <u>without</u> cystic fibrosis?**
   Choose one of the following answers
   ☐ 2dd 2.5 mg by nebulization
   ☐ Other: ______

7. **If yes, for how long is dornase alfa (Pulmozyme) usually prescribed in patients <u>without</u> cystic fibrosis?**
   Choose one of the following answers
   ☐ Single dose
   ☐ One day
   ☐ 1-7 days
   ☐ At least a week
   ☐ Highly variable
   ☐ Guided by resolving of clinical symptoms
   ☐ Other: ______

8. **Do you ever prescribe dornase alfa (Pulmozyme) to patients with non-invasively respiratory support (HFNC/non-invasively ventilated/oxygen mask)?**
   ☐ Yes ☐ No

9. **If yes, what dose regimen do you prescribe to patients with non-invasively respiratory support ?**
   Choose one of the following answers
   ☐ 2dd 2.5 mg by nebulization
   ☐ Other: ____

10. **If yes, for how long is dornase alfa (Pulmozyme) usually prescribed in patients with non-invasively respiratory support?**
    Choose one of the following answers
    ☐ Single dose
    ☐ One day
    ☐ 1-7 days
    ☐ At least a week
    ☐ Highly variable
    ☐ Guided by resolving of clinical symptoms
    ☐ Other: ____

11. **Do you ever prescribe dornase alfa (Pulmozyme) to invasively ventilated patients (endotracheal tube/endotracheal cannula)?**
    ☐ Yes ☐ No

12. **If yes, what dose regimen do you prescribe to invasively ventilated patients?**
    Choose one of the following answers
    ☐ 2dd 2.5 mg by nebulization
    ☐ Other: ____

13. **If yes, for how long is dornase alfa (Pulmozyme) usually prescribed in invasively ventilated patients?**
    Choose one of the following answers
    ☐ Single dose
    ☐ One day
    ☐ 1-7 days
    ☐ At least a week
    ☐ Highly variable
    ☐ Guided by resolving of clinical symptoms
    ☐ Other: ____

14. **Do you ever prescribe dornase alfa (Pulmozyme) as a direct instillation through the endotracheal tube instead of nebulization?**
    ☐ Yes ☐ No

15. **Do you think/know that other fellows/other pediatric intensivists ever prescribe dornase alfa (Pulmozyme) in patients <u>without</u> cystic fibrosis in your PICU?**
    ☐ Yes ☐ No

Current evidence

16. **In your PICU, is there a protocol or guideline as for how use dornase alfa (Pulmozyme) in cystic fibrosis patients to your knowledge?**
    ☐ Yes ☐ No

17. **In your PICU, is there a protocol or guideline as for how use dornase alfa (Pulmozyme) in patients other than cystic fibrosis patients?**
    ☐ Yes ☐ No

18. **In your own experience, do you think dornase alfa (Pulmozyme) is beneficial in selected patients groups in the PICU?**

	No	Sometimes	Yes
**Cystic fibrosis**	O	O	O
**Viral bronchitis**	O	O	O
**Pneumonia**	O	O	O
**Asthma**	O	O	O

19. **Did your patients ever experience side-effects of dornase alfa (Pulmozyme) in your PICU?**
    Check all that apply
    ☐ No
    ☐ Sore and dry throat and hoarseness
    ☐ Eye redness and irritation
    ☐ Rash
    ☐ Laryngitis
    ☐ Allergic reaction (difficulty breathing, swelling lips, tongue or face)
    ☐ Chest pain
    ☐ Other

20. **If yes, in how many patients did you ever experience side-effects of dornase alfa (Pulmozyme) in your PICU? Please estimate.**
    Choose one of the following answers
    ☐ Never
    ☐ 0-5%
    ☐ 5-25%
    ☐ 25-50%
    ☐ 50-100%

Current needs

21. **Do you think research is needed on the effects of dornase Alfa (Pulmozyme) in specific patient groups in the PICU?**
    Choose one of the following answers
    ☐ No
    ☐ Yes, which patient group(s)? [ ]

22. **Do you feel the need for further evidence on the effects of dornase alfa (Pulmozyme) by direct endotracheal instillation as compared to nebulization?**
    ☐ Yes ☐ No

23. In a randomized clinical trial investigating the effect of dornase alfa (Pulmozyme) in invasively ventilated patients in the PICU, what primary outcome would be most suitable to your opinion?
Check all that apply
☐ Mortality
☐ Duration of mechanical ventilation
☐ Length of PICU stay
☐ X-ray confirmed atelectasis
☐ Gas-exchange
☐ Other: ▭

24. In a randomized controlled trial, which of the following questions about dornase alfa (Pulmozyme) prescription should be answered?
Check all that apply
☐ Which dose?
☐ Duration of treatment?
☐ Which criteria to start?
☐ Method of administration?
☐ Other: ▭

Participant characteristics

25. What is your age?
Only numbers may be entered in this field
▭

26. What is your gender?
☐ Female ☐ Male

27. In which PICU are your currently working?
Choose one of the following answers
☐ Amsterdam UMC
☐ Erasmus MC Rotterdam
☐ UMC Leiden
☐ UMC Groningen
☐ UMC Nijmegen
☐ Maastricht UMC
☐ UMC Utrecht

28. For how long are you working as a pediatric intensivist (please include years of fellowship training)?
Choose one of the following answers
☐ <2 years
☐ 2-5 years
☐ 6-15 years
☐ >15 years
☐ Fellowship
