# Supplementary material for: Use of dornase alfa in the paediatric intensive care unit: current literature and a national cross-sectional survey
Source: Eur J Hosp Pharm. 2020 Oct 29;29(3):123–8. doi: 10.1136/ejhpharm-2020-002507 (PMC9047925; doi:10.1136/ejhpharm-2020-002507)
Supplement: Supplementary data [file ejhpharm-2020-002507supp003.pdf]

# Supplemental Table 1

**Summary of study characteristics:**

| Study | Population | Study design | Intervention and control | Outcome(s) of interest |
|---|---|---|---|---|
| Riethmueller et al. 2006 (ref 21) | PICU patients (N=88), post-cardiac surgery | RCT | Dornase alfa versus NaCl 0.9% intra-tracheal instillation, twice daily | - Re-intubation<br>- Atelectasis on chest X-ray<br>- Hours of mechanical ventilation<br>- Length of ICU stay |

**Summary of findings:**

**Dornase alfa compared to normal (0.9%) saline intratracheal instillation**
**Patient or population:** critically ill children
**Setting:** PICU
**Intervention:** Dornase alfa
**Comparison:** Normal (0.9%) saline or placebo

| Outcomes | Population | No of Participants (No of studies) | Control vs intervention | Assumed risk (Mean/SD or Median/IQR) | Corresponding risk (Mean/SD or Median/IQR) | Relative risk (95% CI) | Quality of the evidence (GRADE) | Reference |
|---|---|---|---|---|---|---|---|---|
| | | | | Control | Intervention | | | |
| **Duration of invasive mechanical ventilation (hours)** | PICU | 88 (1 study) | 0.9% vs dornase alfa | 82 (103)[a] | 52 (70)[a] | | ⊕⊕⊕○ [b] Moderate | 21 |
| **Length of ICU stay (hours)** | PICU | 88 (1 study) | 0.9% vs dornase alfa | 192 (216)[a] | 168 (96)[a] | | ⊕⊕⊕○ [b] Moderate | 21 |

| | | | | | | | | |
|---|---|---|---|---|---|---|---|---|
| **Atelectasis on chest X-ray** | PICU | 88 (1 study) | 0.9% vs dornase alfa | 38 per 100 patients | 14 per 100 patients | 0.37 (0.16 to 0.85) | ⊕⊕⊕○ [b] Moderate | 21 |
| **Re-intubation** | PICU | 88 (1 study) | 0.9% vs dornase alfa | 9 per 100 patients | 7 per 100 patients | 0.78 (0.18 to 3.30) | ⊕⊕⊕○ [b] Moderate | 21 |
| **Mortality[c]** | PICU | 88 (1 study) | 0.9% vs dornase alfa | - | - | | | 21 |
| **Respiratory system mechanics[d]** | PICU | 88 (1 study) | 0.9% vs dornase alfa | - | - | | | 21 |
| **Oxygenation/ ventilation indices[d]** | PICU | 88 (1 study) | 0.9% vs dornase alfa | - | - | | | 21 |
| **Adverse events[e]** | PICU | 88 (1 study) | 0.9% vs dornase alfa | 0 per 100 patients | 0 per 100 patients | - | ⊕⊕⊕○ [b] Moderate | 21 |

[a] Interquartile range; 75th percentile minus 25th percentile

[b] The quality of the body of evidence was downgraded due to potential of publication bias/threat to study validity (see Supplemental Table 1).

[c] Mortality was an exclusion criteria in this study.

[d] These outcomes were not reported

[e] No adverse events were seen during this study.



den Hollander B, *et al. Eur J Hosp Pharm* 2022; 29:123–128. doi: 10.1136/ejhpharm-2020-002507
