# Supplementary material for: Use of dornase alfa in the paediatric intensive care unit: current literature and a national cross-sectional survey
Source: Eur J Hosp Pharm. 2020 Oct 29;29(3):123–8. doi: 10.1136/ejhpharm-2020-002507 (PMC9047925; doi:10.1136/ejhpharm-2020-002507)
Supplement: Supplementary data [file ejhpharm-2020-002507supp004.pdf]

Supplemental Table 2 Characteristics of included studies

Riethmueller 2006 (ref 21)

Methods	RCT
Participants	PICU patients (0-2yrs) with invasive mechanical ventilation (expected MV time >24hrs) after cardiac surgery.
Interventions	Dornase alfa (Pulmozyme®) twice daily versus 0.9% saline, by intra-tracheal instillation.
Outcomes	Primary outcome: re-intubation occurrence<br>Secondary outcomes: duration of mechanical ventilation, length of ICU stay, atelectasis resolution or improvement on chest X-ray, re-intubation rate, mortality, respiratory system mechanics measurements (airway pressures and resistance, lung compliance, respiratory system compliance), oxygenation/ventilation indices and adverse events.
Notes	

Risk of bias

**Bias**	**Authors' judgement**	**Support for judgement**
Random sequence generation (selection bias)	Low risk	Stated: "a block randomization list assigning half the sample to each treatment was prepared in advance"
Allocation concealment (selection bias)	Low risk	Stated: "used by the hospital pharmacy in preparation of the study medication"
Blinding of participants and personnel (performance bias)<br>All outcomes	Low risk	Stated: "patients, nurses and physicians were unaware of the nature of the treatment"
Blinding of outcome assessment (detection bias)<br>All outcomes	Low risk	Stated: "radiologist unaware of the treatment"<br>Unknown if researcher was blinded, yet stated "double-blind clinical trial".
Incomplete outcome data (attrition bias)<br>All outcomes	Low risk	No unequal loss of participants was reported
Selective reporting (reporting bias)	Low risk	Given the outcome data it is not probable that this was done

Other bias	High risk	Study sponsored by Hoffman la Roche, manufacturer of dornase alfa. No documentation/registration of study protocol prior to start of study.
